# Accelerated BEP: a phase I trial of dose-dense BEP for intermediate and poor prognosis metastatic germ cell tumour

**DOI:** 10.1038/bjc.2011.309

**Published:** 2011-08-16

**Authors:** Y Rimmer, J Chester, J Joffe, D Stark, J Shamash, T Powles, J White, J Wason, D Parashar, G Armstrong, D Mazhar, M V Williams

**Affiliations:** 1 Oncology Centre, Box 193, Addenbrooke's Hospital, Cambridge University Hospitals NHS Trust, Hills Road, Cambridge CB2 0QQ, UK; 2 St James's Institute of Oncology, Bexley Wing, St James's University Hospital, Beckett Street, Leeds LS9 7TF, UK; 3 Barts and the London Hospitals, Medical Oncology, 7th Floor, Gloucester House, West Smithfield EC1A 7BE, UK; 4 The Beatson West of Scotland Cancer Centre, 1053 Great Western Road, Glasgow G12 0YH, UK; 5 MRC Biostatistics Unit Hub in Trials Methodology Research, Institute of Public Health, University Forvie Site, Robinson Way, Cambridge CB2 0SR, UK; 6 Department of Oncology, Cambridge Cancer Trials Centre, University of Cambridge, Addenbrooke's Hospital (Box 279), Hills Road, Cambridge CB2 0QQ, UK

**Keywords:** accelerated, chemotherapy, growth factors, germ cell tumours, dose-density, bleomycin

## Abstract

**Background:**

We used bleomycin, etoposide, cisplatin (BEP), the most effective regimen in the treatment of germ cell tumours (GCTs) and increased dose-density by using pegfilgrastim to shorten cycle length. Our aim was to assess safety and tolerability.

**Methods:**

Sixteen male patients with intermediate or poor prognosis metastatic GCT were treated with four cycles of 3-day BEP with G-CSF on a 14-day cycle for a planned relative dose-density of 1.5 compared with standard BEP.

**Results:**

Eleven intermediate and five poor prognosis patients were treated. In all, 14 of 16 patients completed the study treatment. Toxicities were comparable to previous studies using standard BEP, except for mucositis and haematological toxicity that were more severe. The overall relative dose-density for all 16 patients was mean 1.38 (range 0.72–1.5; median 1.46). Complete response was achieved after chemotherapy alone in two patients (13%) and following chemotherapy plus surgery in nine additional patients (56%). Four patients (25%) had a partial response and normalised their marker levels. At a median follow-up of 4.4 years (range 2.1–6.8) the estimated 5-year progression-free survival probability is 81% (95% CI 64–100%).

**Conclusion:**

Accelerated BEP is tolerable without major additional toxicity. A randomised controlled trial will be required to obtain comparative efficacy data.

Patients with metastatic germ cell tumours (GCTs) have been classified into three prognostic groups (International Germ Cell Cancer Collaborative Group (IGCCCG), 1997). Despite excellent outcomes in good prognosis patients, 5-year progression-free survival (PFS) rates in intermediate and poor risk patients remain unsatisfactory at 75% and 41%, respectively (International Germ Cell Cancer Collaborative Group (IGCCCG), 1997). For poor prognosis patients treated with bleomycin, etoposide, cisplatin (BEP) in clinical trials, 5-year PFS rates of 44 and 41% were recently confirmed (Motzer *et al*, 2007; Daugaard *et al*, 2010).

Studies initiated before the introduction of the IGCCCG prognostic groups in 1997, included a mixture of intermediate and poor prognosis patients by those criteria (Collette *et al*, 1999; Hinton *et al*, 2003). There have been four main approaches aimed at improving on the results of BEP as first-line therapy for these patients:

1. Intensification of cisplatin dose.
2. Use of new drugs.
3. Sequential alternating drug combinations.
4. Increased drug doses using growth factor or stem cell support.

Over the last 20 years, none of these approaches has been shown to improve outcomes and most have increased toxicity (Nichols *et al*, 1991, 1998; de Wit *et al*, 1998; Kaye *et al*, 1998; Hinton *et al*, 2003). The only exception is the recent publication of an abstract showing a 12% improvement in 3-year PFS for intermediate prognosis patients treated with paclitaxel-BEP (T-BEP) (de Wit *et al*, 2011). This may be the first improvement in outcome since the development of BEP 25 years ago (Williams *et al*, 1987) and full publication is awaited.

Higher drug doses can be given with growth factor support. Increased doses of cisplatin and etoposide (Daugaard and Rørth, 1986) and ifosphamide and etoposide (Schmoll *et al*, 2003) have been achieved but toxicity was unacceptable. Subsequently stem cell support was used to deliver high-dose VIP with a cycle interval of 21 days: over 5 days, the dose of etoposide was escalated four-fold (1500 mg m^–2^), that of ifosphamide was doubled (12 g m^–2^) and cisplatin was given in standard dose (100 mg m^–2^). The regimen was more toxic and no more effective than BEP in a randomised study of 137 patients (Daugaard *et al*, 2010). High-dose therapy as first-line management has been tested in a clinical trial of 219 patients: four cycles of BEP were compared with two cycles followed by two high-dose procedures each with stem cell autograft. There was no improvement in 1-year durable complete response (CR) rate compared with BEP (Motzer *et al*, 2007).

By contrast, dose-dense regimens increase dose-exposure by shortening inter-treatment intervals rather than by increasing doses. It has been hypothesised that delivering the same total drug dose in the same number of cycles but in a shorter time should improve the impact of therapy (Citron *et al*, 2003; Fornier and Norton, 2005). Studies in a variety of tumours have shown that relapse-free and overall survival can be improved but the results are inconsistent (Fizazi and Zelek, 2000; Thatcher *et al*, 2000; Ardizzoni *et al*, 2002; Citron *et al*, 2003; Pfreundschuh *et al*, 2004; Lorigan *et al*, 2005; Bonilla *et al*, 2010).

We planned to increase the dose-density of BEP, the most effective regimen in the treatment of GCTs, by using pegfilgrastim to shorten the cycle length. This is a novel approach in GCTs and our primary aim was to assess safety and tolerability.

## Patients and methods

### Patients

Ethical approval was granted by the UK's Eastern Multi-Centre Research Ethics Committee (EudraCT number: 2004–000847–79, ISRCTN18505589) and all patients gave written informed consent. Patients were eligible for this study if they had metastatic GCT with either intermediate or poor prognosis disease (International Germ Cell Cancer Collaborative Group (IGCCCG), 1997). Histological confirmation of GCT was desirable but not essential providing *α*-foetoprotein >1000 ng ml^–1^ and/or *β-*human chorionic gonadotrophin (*β*-HCG) >5000 IU l^–1^.

Male patients with no history of previous malignancy (except basal cell carcinoma of the skin) nor prior treatment with chemotherapy or radiotherapy were eligible. No induction treatment was permitted before BEP. Patients were excluded if creatinine clearance was below 60 ml min^–1^ (unless secondary to obstructive uropathy, which could be relieved by nephrostomy) or if neutrophils <1.0 × 10^9^ l^–1^ and platelets <100 × 10^9^ l^–1^ before the start of treatment.

Patients were staged with CT scan of brain, chest, abdomen and pelvis. Bone scan was undertaken if clinically indicated by symptoms or elevated alkaline phosphatase. Audiometry, lung function tests and EDTA clearance were undertaken before, and on completion of treatment. Neurotoxicity was assessed by clinical examination and using a structured patient questionnaire at baseline and at intervals thereafter (Cassidy *et al*, 1998).

### Treatment

The protocol was based on a 3-day version of BEP, which delivers the same total drug doses as standard BEP (de Wit *et al*, 2001; Fossa *et al*, 2003). The accelerated BEP regimen consisted of four cycles of 3-day BEP with an intended cycle interval of 14 days. Cisplatin 50 mg m^–2^ i.v. was administered on days 1 and 2 and etoposide 165 mg m^–2^ i.v. on days 1, 2 and 3. Bleomycin infusions (30 000 units) were administered on day 2 and between days 6–8 and 10–12. Pegfilgrastim (Neulasta) 6 mg was given subcutaneously on day 4 of each cycle. The planned elapsed time was therefore 8 weeks rather than 12 weeks for a planned relative dose-density of 1.5 for each drug compared with standard BEP.

Subsequent cycles of chemotherapy were scheduled to commence on day 15 and were given if the neutrophil count was ⩾1.0 × 10^9^ l^–1^ and the platelet count was ⩾50 × 10^9^ l^–1^. If these levels were not achieved, counts were repeated at 24-h intervals until the criteria were met. If there were grade 3 or 4 mucosal toxicity, diarrhoea or skin toxicity, then chemotherapy was delayed until recovery. Patients with a delay of a week or longer were withdrawn from the study and completed treatment with the standard (3-weekly) BEP regimen.

### Serious adverse event reporting and stopping rules

Toxicity severity was reported according to the Common Terminology Criteria for Adverse Events (CTCAE) v3.0 (Cancer Therapy Evaluation Program, 2006). The following were specified as serious adverse events (SAEs) in this trial and also comprised part of the stopping rules:

- Life-threatening bleeding refractory to platelet support.
- Life-threatening sepsis with septic shock.
- Impairment of renal function with a reduction in clearance below 60 ml min^–1^.
- Death on treatment from any cause.
- Fatal bleomycin pulmonary toxicity.

An incidence of these specified events of >15% would have been considered unacceptable. Patients were entered in cohorts of 5, 5 and 6 and toxicity was assessed by an independent data monitoring committee. Trial entry was suspended between each cohort until each patient had completed planned chemotherapy and post-treatment assessment (9–10 weeks after entry).

### Bleomycin toxicity

Close clinical monitoring was used to assist in weighing the risks and benefits of each dose of bleomycin for the individual patient (Saxman *et al*, 1997; Nichols *et al*, 1998; O’Sullivan *et al*, 2003). Patients were questioned regarding respiratory symptoms and examined for basal crepitations before each dose of bleomycin. Chest radiographs were serially reviewed every 15 days immediately before each cycle to detect abnormalities suggestive of an interstitial process (Saxman *et al*, 1997).

### Response evaluation

Response was assessed in all patients within 3 to 4 weeks of the start of the final cycle of therapy (i.e., weeks 9 to 10 of the study) with CT scan of chest, abdomen and pelvis and repeat of any other investigation abnormal at the outset. In addition, standard blood tests (including tumour markers), Cr^51^-EDTA clearance, audiometry, pulmonary vital capacity, neurotoxicity questionnaire and clinical assessment were performed.

Patients with residual masses (abdominal or pulmonary lesions >1 cm) post-treatment were considered for surgical resection within 6–8 weeks. If markers were still decreasing, continued observation was considered appropriate. If there was a significant rise in markers (doubling or two consecutive rises), the patient was defined as a treatment failure eligible for salvage chemotherapy at the investigator's discretion.

After completion of treatment, patients were followed up according to institutional practice. CT of chest, abdomen and pelvis was performed 2 years after completion of treatment. Disease progression was defined as a consistent increase in tumour markers (at least two measurements 1–2 weeks apart) or clinical or radiological evidence of an increase in the size of residual lesions or occurrence of new lesions.

### Statistical design

This study was a multi-centre, non-randomised, non-controlled phase I study. The primary objectives were to determine the feasibility of the regimen and to determine toxicity particularly with respect to renal, pulmonary and neurological function. The secondary objectives were to estimate the response rate and PFS using a Kaplan–Meir estimator.

To assess drug delivery, we calculated dose-density for each drug as the product of two quantities: the ratio of days taken by the existing BEP regimen to days taken by the accelerated regimen; and the proportion of planned doses given. We set no study target and a patient who completed the accelerated regimen within the planned time and received all planned doses had a dose-density of 1.5. As bleomycin may be discontinued prematurely to minimise the risk of toxicity in 5–30% of patients receiving standard BEP (Saxman *et al*, 1997; Nichols *et al*, 1998), patients who stopped bleomycin early had their dose-density calculated at the day of final dose.

## Results

### Patients

Between August 2004 and February 2009, 16 patients (11 with intermediate prognosis and 5 with poor prognosis) non-seminoma GCT were recruited from four UK centres. The median age of the cohort was 29 years (range 20–41). The baseline characteristics of the patients and their response to therapy are shown Table 1. There were no treatment related deaths.

### Dose-density of regimen

A total of 14 of 16 patients completed all four planned cycles within the trial. In all, 12 of these 14 (75% of all patients) completed the accelerated regimen with full doses of all three drugs (Table 2). The remaining two patients received full doses of cisplatin and etoposide, but with early cessation of bleomycin (see below). Overall 13 out of 16 (81%) patients commenced the fourth cycle within 1 week of schedule, and 12 of these completed their cisplatin and etoposide with total delays of no more than 5 days. Relative dose-density for all 16 patients was cisplatin 1.37 (range 0.49–1.5), etoposide 1.37 (0.5–1.5) and bleomycin 1.40 (0.98–1.5) for an overall relative dose-density of 1.38 (range 0.72–1.5; median 1.46).

### Withdrawal from study

Two patients were withdrawn from the study after cycle 2 because of toxicity. Both completed chemotherapy using 3-weekly cycles. They remain alive and progression-free and are included in the response analysis.

Patient 002 was admitted on day 7 of cycle one with grade 2 mucositis, grade 3 neutropaenic sepsis and grade 4 thrombocytopaenia. In cycle 2, the same toxicity occurred again and he was withdrawn from the study. He received a further cycle of BEP (after an interval of 24 days, rather than the planned 14 days). Bleomycin was stopped electively because of patient frailty (no pulmonary toxicity was reported) and he received a final cycle of EP. Both these cycles were again complicated by neutropaenic sepsis. He was a complete responder to chemotherapy and surgery.

Patient 007 was withdrawn from the study after admission on day 12 of cycle 2 with grade 3 mucositis and grade 4 neutropaenia and thrombocytopaenia. During this episode he missed a single dose of bleomycin. He subsequently received two cycles of standard 3-weekly BEP. No pulmonary toxicity was reported. He was a complete responder to chemotherapy and surgery.

### Haematological toxicity

Table 3 shows the highest toxicity score recorded during the study chemotherapy for each patient. Overall there were 27 SAEs reported, including nine reports of admission for neutropaenic sepsis and two for the management of thrombocytopaenia. The percentage of patients with grade 3/4 haematological toxicity recorded during at least one cycle was: thrombocytopaenia 56%, neutropaenia 63% and febrile neutropaenia 38%. Four patients received platelet support and nine received red cell transfusions.

### Renal toxicity

Four patients had grade 1 toxicity and one grade 2 toxicity (Table 3). EDTA clearance was available at baseline and 8 weeks for 12 out of 14 patients who completed the protocol. Mean renal function at baseline was 130 ml min^–1^ (range 88–208) and at 9–10 weeks the mean was 84 ml min^–1^ (range 56–136) a mean reduction to 66% of baseline (range 50–89%).

### Pulmonary toxicity

Table 3 shows that grade 3/4 pulmonary symptoms were reported for a single patient who had disease related dyspnoea (grade 3), hypoxia (grade 2) and cough (grade 1). These symptoms all improved towards the end of chemotherapy and he received full dose bleomycin.

Table 2 shows that bleomycin was delivered within 7 days of schedule and in full dose to 12 out of 16 patients. Two patients were withdrawn from the study because of non-pulmonary toxicity (see above). One patient missed the final dose of bleomycin and was recorded as having a cough but no dyspnoea or other abnormalities. He made a full recovery. One patient received only 180 000 units of bleomycin (six doses) because of grade 1 cough and grade 1 dyspnoea. He made a full recovery.

Lung function data were available on eight patients. At baseline the mean lung diffusing capacity for carbon monoxide (DLCO) was 93% of predicted (range 60–119%). At the completion of treatment, this showed a reduction to a mean of 66% of expected (range 45–79%). This reduction in DLCO affected all patients and there was a mean reduction to 73% of the baseline value (range 57–83%). Five patients had long-term follow-up data and all showed improvement.

### Neurological toxicity

Table 3 shows that 10 patients had no recorded neurotoxicity. Neurological assessment was reported at 8 weeks on 12 patients of whom 4 were normal and 6 had objective evidence of a grade 1 sensory deficit: this recovered in all but a single patient who had grade 2 signs at 10 months. Two patients had grade 2 sensory loss (one with grade 2 motor signs in the hands): both recovered on later assessments.

Patient questionnaires were returned for the 8-week assessment on 11 patients of whom 4 had symptoms all of which improved on follow-up so that only 1 patient still had troubling symptoms at most recent follow-up.

### Auditory toxicity

Three of 14 patients had grade 2 auditory toxicity, defined as hearing loss not requiring intervention (Table 3). Mild tinnitus not interfering with the activities of daily living (grade 2) was reported by four patients. Pre- and post-treatment audiograms were available on 11 patients of whom 6 showed no change. Five showed substantial high-tone loss at 8 Hz with a threshold of 40–70 dB. Of the three cases with longer follow-up, two showed full recovery on audiograms at 1 and 3 years. Thus, 8 out of 11 (72%) had objectively normal hearing in the long term.

### Other toxicity

Mucositis was common with 50% of patients experiencing grade 2 symptoms and 25% experiencing grade 3 symptoms during at least one cycle of their treatment (Table 1). Three patients had transitory grade 3 dysphagia. Two patients were treated for hypomagnesaemia.

### Response analysis

All 16 patients were included in the response analysis (see Table 1). The overall objective response rate was 94%. Complete response with normal radiology and tumour markers was achieved in 11 out of 16 patients (69%) – after chemotherapy alone in 2 patients (13%) and following surgery in 9 patients (56%). As no viable cancer cells were detected in any of these specimens, these patients were all classified as complete responders. A further four patients (25%) had a partial response (PR) and normalised their marker levels. This included one patient with small foci of immature teratoma with sarcomatoid change. He also had residual lung metastases that progressed (see Table 1). One patient was classified as a treatment failure because of increasing tumour markers within 7 weeks of completion of accelerated BEP, having never normalised his *β*-HCG levels. Salvage treatment was at the investigator's discretion and is summarised in Table 1.

### Progression-free survival

**Figure 1:**
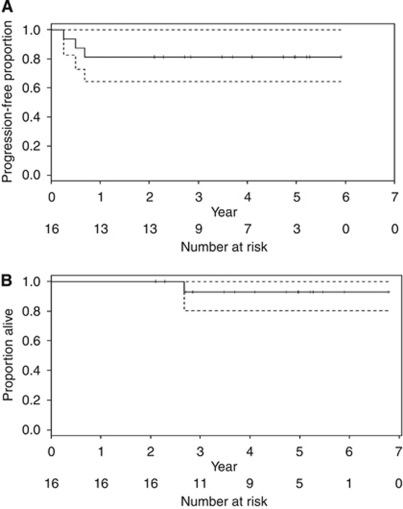
Kaplan–Meier survival curve with upper and lower 95% confidence bands (dashed). The estimated 5-year PFS probability is 81% (95% CI 64–100%) (**A**). The estimated 5-year overall survival probability is 92% (95% CI 77–100%) (**B**).

Median follow-up was 4.4 years (range 2.1–6.8). Three patients have experienced progression since treatment and one of these has died because of progressive disease (see Table 1). Figure 1 shows that the estimated 5-year PFS probability is 81% (95% CI 64–100%) (panel A). The estimated 5-year overall survival probability is 92% (95% CI 77–100%) (panel B).

## Discussion

This phase I study showed that accelerated BEP does not produce significant additional toxicity, compared with the standard 3-weekly regimen, particularly with respect to renal function and pulmonary toxicity. Accelerated BEP causes more mucositis than standard BEP with 50% of patients having grade 2 toxicity on at least one occasion and 25% grade 3. This contributed to the withdrawal of two patients from the study treatment. The haematological toxicity was similar to that reported with VIP, BOP/VIP-B and CBOP/BEP (Kaye *et al* 1998; Nichols *et al*, 1998; de Wit *et al* 2001; Christian *et al* 2003; Fossa *et al*, 2005) but greater than that reported with 3-weekly BEP (de Wit *et al* 2001), even when used to treat intermediate and high-risk patients (Kaye *et al* 1998; Nichols *et al* 1998).

Standard BEP delivering cisplatin 400 mg m^–2^ causes renal injury with a reduction to 77–89% of the baseline clearance measurement (Macleod *et al* 1988; Hamilton *et al* 1989; Bissett *et al*, 1990). Previous studies that increased the dose-density or dose-intensity of cisplatin caused increased nephrotoxicity: double dose cisplatin reduced renal function to 68% of baseline (Daugaard and Rørth, 1986); CBOP/BEP caused a mean reduction of EDTA clearance to 61 and 70% of baseline in two studies (Horwich *et al*, 1989, 1994). Accelerated BEP in our study reduced mean renal function to 66% of baseline. This toxicity is greater than that of standard BEP, but is comparable to that of other intensive regimens and is not dose-limiting.

Bleomycin pulmonary toxicity was a major concern in the planning phase of this study. The administration of standard BEP involves the delivery of bleomycin 360 000 units in 12, weekly intravenous doses of 30 000 units: the landmark study that established BEP as the standard of care reported fatal bleomycin toxicity in 2% of cases (Williams *et al*, 1987). Increased dose-density of bleomycin has been explored in the development of CBOP/BEP. The original version of this regimen delivered 360 000 units over 4 weeks and a total dose of 450 000 units. This was associated with fatal toxicity in 3 of 15 patients (Horwich *et al*, 1989). The current CBOP/BEP regimen delivers 180 000 units over 4 weeks in the induction protocol with a further 11-weekly doses of bleomycin 15 000 units to a total dose of 345 000 units with acceptable pulmonary toxicity (Christian *et al*, 2003; Fossa *et al*, 2005). The accelerated BEP protocol replicates the bleomycin dose-density of the induction protocol but continues it for 8 weeks (to a total dose of 360 000 units). We found that the increased mean dose-density of bleomycin of 1.40 (range 0.98–1.5; median 1.49) relative to standard BEP was tolerable. The reduction in DLCO was consistent with previous studies and as expected showed improvement on follow-up (Osanto *et al*, 1992). Bleomycin was discontinued in two patients after 180 000 and 330 000 units because of symptoms suggestive of toxicity but both made a full recovery. Previous studies have reported premature discontinuation of bleomycin to minimise the risk of toxicity in 5–30% of patients receiving standard BEP (Saxman *et al*, 1997; Nichols *et al*, 1998). The reported incidence of bleomycin toxicity is 3–5% with a death rate of 1–2% (Williams *et al*, 1987; Kaye *et al*, 1998; Nichols *et al*, 1998; O’Sullivan *et al* 2003). Our results are consistent with previous findings but the study is too small to exclude an increased risk.

We used a 3-day version of BEP, which delivers the same total drug doses as standard BEP (de Wit *et al*, 2001; Fossa *et al*, 2003), but in 8 weeks, rather than 12. The 3-day regimen has only been assessed previously in good prognosis patients, where it was as effective as 5-day BEP, when given at 3-weekly intervals (de Wit *et al*, 2001), but caused increased tinnitus and gastrointestinal toxicity over four cycles (Fossa *et al*, 2003). The 3-day regimen was chosen to permit the administration of Pegfilgrastim (Neulasta) 6 mg on day 4 and to allow an 11 day interval without myelosuppressive drugs.

While our study was being conducted an Australian study of accelerated BEP using a 5-day BEP schedule given every 2 weeks was opened for patients in all prognostic groups (Grimison *et al*, 2011). Bleomycin was administered at weekly intervals and therefore continued after administration of the other drugs had been completed. An interim report states that toxicity was acceptable with 36 out of 41 (88%) patients eligible to start a fourth cycle of treatment within 1 week of schedule (Grimison *et al*, 2011); in our study this was 13 out of 16 patients (81%). They report that 1-year PFS was 80% for 25 patients with an intermediate or poor prognosis (Grimison *et al*, 2011). Our study with a group of 16 patients of a similarly mixed prognosis showed an estimated 5-year PFS probability of 81% (95% CI 64–100%). As the data from both studies mature, combined analysis may give a better estimate of efficacy to assist in considering the case for a randomised trial.

## Conclusion

Accelerated BEP is a novel, dose-dense regimen that was tolerable for the majority of our patients. It shares with T-BEP (de Wit *et al*, 2011) the simple concept of intensifying standard BEP. Mucositis and haematological toxicity were somewhat increased. Renal toxicity was comparable to that of other intensive regimens. Neurological and auditory toxicity were acceptable. The sample size was too small to exclude a change in the incidence of bleomycin pulmonary toxicity, which is a rare event. We conclude that accelerated BEP merits further evaluation in terms of efficacy: this would require a multinational randomised controlled trial.

## Figures and Tables

**Table 1:** Patient baseline characteristics and response to therapy

**Trial no.**	**Age (at enrolment)**	**Prognostic category (IGCCCG classification)**	**Response to chemotherapy**	**Surgery**	**Surgical histology**	**Overall response**	**Relapse**	**Comment**
001	41	Poor	Progression	None		Progression	Progression	
002	20	Poor	PR marker neg	RPLND	No viable tumour	CR chemo and surgery		Withdrawn
003	21	Intermediate	PR marker neg	RPLND	No viable tumour	CR chemo and surgery		
004	22	Intermediate	PR marker neg	RPLND	No viable tumour	CR chemo and surgery		
005	30	Intermediate	PR marker neg	None		PR marker neg	Relapse	
006	37	Poor	PR marker neg	RPLND	No viable tumour	CR chemo and surgery		
007	35	Intermediate	PR marker neg	Node biopsy	No viable tumour	PR marker neg		Withdrawn
008	23	Intermediate	PR marker neg	RPLND	No viable tumour	CR chemo and surgery		
009	29	Intermediate	PR marker neg	None		PR marker neg		
010	29	Intermediate	PR marker neg	RPLND	Foci of viable tumour (sarcomatoid change)	PR marker neg	Relapse	Died tumour
011	26	Intermediate	PR marker neg	RPLND	No viable tumour	CR chemo and surgery		
012	21	Intermediate	Complete remission	None		CR chemotherapy alone		
013	33	Poor	PR marker neg	Orchidectomy	No viable tumour	CR chemo and surgery		
014	29	Poor	PR marker neg	RPLND	No viable tumour	CR chemo and surgery		
015	33	Intermediate	PR marker neg	RPLND	No viable tumour	CR chemo and surgery		
016	28	Intermediate	Complete remission	None		CR chemotherapy alone		

Abbreviations: CR=complete response; neg=negative; PR=partial response; RPLND=retroperitoneal lymph node dissection. Salvage therapy: patient 001: Granulocyte colony stimulating factor (GCSF), actinomycin D, methotrexate, etoposide, cisplatin (GAMEC) chemotherapy (Shamash *et al*, 2007b) followed by RPLND showing no viable tumour. Remains progression free 6 years later.

Patient 005: GAMEC chemotherapy (Shamash *et al*, 2007b) followed by RPLND showing no viable tumour. Relapsed in abdomen. Response to irinotecan, paclitaxel, oxaliplatin (IPO) chemotherapy (Shamash *et al*, 2007a); followed by high-dose thiotepa, topotecan, carboplatin with stem cell reinfusion (Shamash *et al*, 2007a). Radiotherapy to para-aortic nodes. Remains progression free 4 years later.

Patient 010: RPLND and orchidectomy included small foci of immature teratoma with sarcomatoid change. Progression of lung metastases 3 months later. VIDE chemotherapy (Ladenstein *et al*, 2010) – progressed. Response to IPO (Shamash *et al*, 2007a); followed by high dose thiotepa, topotecan, carboplatin with stem cell reinfusion (Shamash *et al*, 2007a). Progressed after 9 months. Died of disease.

**Table 2:** Drug delivery and dose-density

		**Bleomycin**			**Etoposide**			**Cisplatin**			**Overall**	
		**Delivered total drug dose (IU)**	**Overall time (days to final dose)**	**Relative dose-density**	**Delivered total drug dose mg** **m^–2^**	**Overall time (days to final dose)**	**Relative dose-density**	**Delivered total drug dose mg** **m^–2^**	**Overall time (days to final dose)**	**Relative dose-density**	**Days to start cycle 4**	**Mean relative dose-density**
**Trial no.**	**Target dose and time**	**360 000**	**52–54**	**1.50**	**2000**	**44**	**1.50**	**400**	**43**	**1.50**	**42**	**1.50**
001		360 000	52	1.50	2000	44	1.50	400	43	1.50	42	1.50
002		240 000	46	1.17	2000	132	0.50	400	131	0.49	130	0.72
003		360 000	53	1.50	2000	44	1.50	400	43	1.50	42	1.50
004		360 000	52	1.50	2000	44	1.50	400	43	1.50	42	1.50
005		360 000	52	1.50	2000	44	1.50	400	43	1.50	42	1.50
006		360 000	52	1.50	2000	44	1.50	400	43	1.50	42	1.50
007		330 000	76	0.98	2000	64	1.03	400	63	1.02	62	1.01
008		360 000	53	1.50	2000	45	1.47	400	44	1.47	43	1.48
009		360 000	58	1.40	2000	51	1.29	400	50	1.29	49	1.33
010		360 000	55	1.47	2000	48	1.38	400	47	1.37	46	1.41
011		360 000	61	1.33	2000	44	1.50	400	43	1.50	42	1.44
012		360 000	54	1.50	2000	44	1.50	400	43	1.50	42	1.50
013		330 000	55	1.35	2000	49	1.35	400	48	1.34	47	1.35
014		180 000	30	1.35	2000	44	1.50	400	43	1.50	42	1.45
015		360 000	60	1.35	2000	52	1.27	400	51	1.26	50	1.29
016		360 000	53	1.50	2000	44	1.50	400	43	1.50	42	1.50
												
*All patients*												
Mean		337 500	53.9	1.40	2000	51.8	1.37	400	50.8	1.37	49.8	1.38
Range		(180–360)	(30–76)	(0.98–1.5)	(2000–2000)	(44–132)	(0.5–1.5)	(400–400)	(43–131)	(0.49–1.5)	(42–130)	(0.72–1.5)
												
*Excluding 002, 007*												
Mean		345 000	52.9	1.45	2000	45.8	1.45	400	44.8	1.45	43.8	1.45
Range		(180–360)	(30–61)	(1.33–1.5)	(2000–2000)	(44–52)	(1.27–1.5)	(400–400)	(43–51)	(1.26–1.5)	(42–50)	(1.29–1.5)

Notes on bleomycin dosage.

Patient 002: patient withdrawn. Bleomycin discontinued electively. No record of pulmonary symptoms or toxicity.

Patient 007: patient withdrawn. One dose of bleomycin omitted. No record of pulmonary symptoms or toxicity.

Patient 013: pulmonary symptoms. Bleomycin stopped. Full recovery (see text).

Patient 014: pulmonary symptoms. Bleomycin stopped. Full recovery (see text).

**Table 3:** Highest toxicity scores for each patient during chemotherapy

	**Grade 0**	**1**	**2**	**3**	**4**
*Haematological*					
Platelets	5	1	1	5	4
Neutropaenia	4	2	0	3	7
Febrile neutropaenia	9	1	0	6	0
					
*Transfusion*					
Platelets	12	4			
Red cells	7	9			
					
*Pulmonary*					
Cough	6	10			
Dyspnoea	10	3	2	1	
Hypoxia	13	0	3		
Pulmonary infiltrates	16				
					
*Renal*					
Creatinine	11	4	1		
					
*Gastrointestinal*					
Nausea	0	10	4	2	
Vomiting	2	4	10		
Diarrhoea	13	1	1	1	
Constipation	8	7	1		
Mucositis	2	2	8	4	
Dysphagia	11	2	0	3	
Skin rash	8	4	2	1	1
					
*Neuropathy*					
Sensory	10	4	2		
Muscle weakness	14	1	1		
Hearing	8	5	3		
					
*Other*					
Fatigue	1	6	7	2	

## References

[bib1] Ardizzoni A, Tjan-Heijnen VCG, Postmus PE (2002) Standard versus intensified chemotherapy with granulocyte colony-stimulating factor support in small-cell lung cancer: a prospective European organisation for research and treatment of cancer – lung Cancer Group phase III trial–08923. J Clin Oncol 20: 3947–3955 12351591 10.1200/JCO.2002.02.069

[bib2] Bissett D, Kunkeler L, Zwanenburg L, Paul J, Gray C, Swan IR, Kerr DJ, Kaye SB (1990) Long-term sequelae of treatment for testicular germ cell tumours. Br J Cancer 62: 655–659 2171622 10.1038/bjc.1990.350 PMC1971502

[bib3] Bonilla L, Ben-Aharon I, Vidal L, Gafter-Gvili A, Leibovici L, Stemmer SM (2010) Dose-dense chemotherapy in nonmetastatic breast cancer: a systematic review and meta-analysis of randomized controlled trials. J Natl Cancer Inst 102: 1845–1854 21098761 10.1093/jnci/djq409 PMC3001963

[bib4] Cancer Therapy Evaluation Program, National Cancer Institute (2006) Common Terminology Criteria for Adverse Events version 3.0 (CTCAE). http://ctep.cancer.gov/protocolDevelopment/electronic_applications/docs/ctcaev3.pdf

[bib5] Cassidy J, Paul J, Soukop M, Habeshaw T, Reed NS, Parkin D, Kaye SB (1998) Clinical trials of nimodipine as a potential neuroprotector in ovarian cancer patients treated with cisplatin. Cancer Chemother Pharmacol 41: 161–166 9443630 10.1007/s002800050723

[bib6] Christian JA, Huddart AR, Norman A (2003) Intensive induction chemotherapy with CBOP/BEP in patients with with poor prognosis germ cell tumours. J Clin Oncol 21: 871–877 12610187 10.1200/JCO.2003.05.155

[bib7] Citron ML, Berry DA, Cirrincione C, Hudis C, Winer EP, Gradishar WJ, Davidson NE, Martino S, Livingston R, Ingle JN, Perez EA, Carpenter J, Hurd D, Holland JF, Smith BL, Sartor CI, Leung EH, Abrams J, Schilsky RL, Muss HB, Norton L (2003) Randomised trials of dose-dense versus conventionally scheduled and sequential (2003) versus concurrent combination chemotherapy as postoperative adjuvant treatment of node-positive primary breast cancer: first report of inter-group trial C 9741/Cancer and Leukemia group B trial 9471. J Clin Oncol 21: 1431–1439 12668651 10.1200/JCO.2003.09.081

[bib8] Collette L, Sylvester RJ, Stenning SP, Fossa SD, Mead GM, de Wit R, de Mulder PH, Neymark N, Lallemand E, Kaye SB (1999) Impact of the treating institution on survival of patients with “poor-prognosis” metastatic nonseminoma. European Organization for Research and Treatment of Cancer Genito-Urinary Tract Cancer Collaborative Group and the Medical Research Council Testicular Cancer Working Party. J Natl Cancer Inst 91: 839–846 10340903 10.1093/jnci/91.10.839

[bib9] Daugaard G, Rørth M (1986) High-dose cisplatin and VP-16 with bleomycin, in the management of advanced metastatic germ cell tumours. Eur J Cancer Clin Oncol 22: 477–485 2426115 10.1016/0277-5379(86)90115-x

[bib10] Daugaard G, Skoneczna I, Aass N, De Wit R, De Santis M, Dumez H, Marreaud S, Collette L, Lluch JR, Bokemeyer C, Schmoll HJ (2010) A randomized phase III study comparing standard dose BEP with sequential high-dose cisplatin, etoposide, and ifosfamide (VIP) plus stem-cell support in males with poor-prognosis germ-cell cancer. An intergroup study of EORTC, GTCSG, and Group Germinal (EORTC 30974). Ann Oncol 22: 1054–1061 21059637 10.1093/annonc/mdq575 PMC3082158

[bib11] de Wit R, Roberts JT, Wilkinson PM, de Mulder PH, Mead GM, Fosså SD, Cook P, de Prijck L, Stenning S, Collette L (2001) Equivalence of 3 or 4 cycles of bleomycin, etoposide and cisplatin chemotherapy and of a 3-day or 5-day schedule in good-prognosis germ cell cancer: a randomised study of the European organisation for research and treatment of cancer. Genito-Urinary Tract Cancer Co-operative Group and the Medical Research Council. J Clin Oncol 19: 1629–1640 11250991 10.1200/JCO.2001.19.6.1629

[bib12] de Wit R, Skoneczna IA, Daugaard KG, de Santis M, Garin A, Aass N, Witjes JA, Albers P, White J, Germa-Lluch JR, Osanto S, Marreaud S, Collette L (2011) A randomized phase III study comparing paclitaxel-BEP (T-BEP) to standard BEP in patients with in intermediate prognosis germ cell cancer (GCC): an intergroup study of EORTC, German TCSG/AUO, MRC, and Spanish GCC group (EORTC 30983). Ann Oncol 22(5): 1054–1061. ASCO abstract 450921059637

[bib13] de Wit R, Stoter G, Sleijfer DT, Neijt JP, ten Bokkel Huinink WW, de Prijck L, Collette L, Sylvester R (1998) Four cycles of BEP *vs* four cycles of VIP in patients with intermediate-prognosis metastatic testicular non-seminoma: a randomized study of the EORTC Genitourinary Tract Cancer Cooperative Group. European Organization for Research and Treatment of Cancer. Br J Cancer 78: 828–832 9743309 10.1038/bjc.1998.587 PMC2062963

[bib14] Fizazi K, Zelek L (2000) Is ‘one cycle every three or four weeks’ obsolete? A critical review of dose-dense chemotherapy in solid neoplasms. Ann Oncol 11: 133–149 10761747 10.1023/a:1008344014518

[bib15] Fornier M, Norton L (2005) Dose-dense adjuvant chemotherapy for primary breast cancer. Breast Cancer Res 7: 64–69 15743513 10.1186/bcr1007 PMC1064124

[bib16] Fossa SD, de Wit R, Roberts JT, Wilkinson PM, de Mulder PH, Mead GM, Cook P, de Prijck L, Stenning S, Aaronson NK, Bottomley A, Collette L; European Organization for Research and Treatment of Cancer Genitourinary Group 30941; Medical Research Council Testicular Cancer Study Group TE20 (2003) Quality of life in good prognosis patients with metastatic germ cell cancer: a prospective study of the European Organization for Research and Treatment of Cancer Genitourinary Group/Medical Research Council Testicular Cancer Study Group (30941/TE20). J Clin Oncol 21: 1107–1118 12637478 10.1200/JCO.2003.02.075

[bib17] Fossa SD, Paluchowska B, Horwich A, Kaiser G, de Mulder PH, Koriakine O, van Oosterom AT, de Prijck L, de Wit R, EORTC GU Group (2005) Intensive induction chemotherapy with C-BOP/BEP for intermediate- and poor-risk metastatic germ cell tumours (EORTC trial 30948). Br J Cancer 93: 1209–1214 16251877 10.1038/sj.bjc.6602830 PMC2361516

[bib18] Grimison PS, Thomson DB, Stockler M, Chatfield MD, Friedlander M, Gebski V, Boland AL, Houghton BB, Gurney H, Rosenthal M, Singhal N, Kichenadasse G, Wong SS, Lewis CR, Vasey PA, Toner GC (2011) Accelerated BEP for advanced germ cell tumors: an australian multicenter phase I/II trial. J Clin Oncol (2011 ASCO Annu Meet Proc (Post-Meeting Edition)) 29(15 Suppl): 4561.

[bib19] Hamilton CR, Bliss JM, Horwich A (1989) The late effects of Cis-platinum on renal function. Eur J Cancer Clin Oncol 25: 185–189 2467807 10.1016/0277-5379(89)90006-0

[bib20] Hinton S, Catalano PJ, Einhorn LH, Nichols CR, David Crawford E, Vogelzang N, Trump D, Loehrer Sr PJ (2003) Cisplatin, etoposide and either bleomycin or ifosfamide in the treatment of disseminated germ cell tumors: final analysis of an intergroup trial. Cancer 97: 1869–1875 12673712 10.1002/cncr.11271

[bib21] Horwich A, Brada M, Nicholls J (1989) Intensive induction chemotherapy for poor risk non-seminomatous germ cell tumours. Eur J Cancer 25: 177–184 10.1016/0277-5379(89)90005-9 2467806

[bib22] Horwich A, Dearnaley DP, Norman A, Nicholls J, Hendry WF (1994) Accelerated chemotherapy for poor prognosis germ cell tumours. Eur J Cancer 30A: 1607–1611 7530470 10.1016/0959-8049(94)00329-4

[bib23] International Germ Cell Cancer Collaborative Group (IGCCCG) (1997) International germ cell consensus classification: a prognosis factor-based staging system for metastatic germ cell cancers. J Clin Oncol 15: 594–603 9053482 10.1200/JCO.1997.15.2.594

[bib24] Kaye SB, Mead GM, Fossa S, Cullen M, deWit R, Bodrogi I, van Groeningen C, Sylvester R, Collette L, Stenning S, De Prijck L, Lallemand E, deMulder P (1998) Intensive induction-sequential chemotherapy with BOP/VIP-B compared with treatment with BEP/EP for poor prognosis metastatic nonseminomatous germ cell tumour: a randomised Medical Research Council/European Organisation for Research and Treatment of Cancer Study. J Clin Oncol 16: 692–701 9469359 10.1200/JCO.1998.16.2.692

[bib25] Ladenstein R, Pötschger U, Le Deley MC, Whelan J, Paulussen M, Oberlin O, van den Berg H, Dirksen U, Hjorth L, Michon J, Lewis I, Craft A, Jürgens H (2010) Primary disseminated multifocal Ewing sarcoma: results of the Euro-EWING 99 trial. J Clin Oncol 28: 3284–3291 20547982 10.1200/JCO.2009.22.9864

[bib26] Lorigan P, Woll PJ, O’Brien ME, Ashcroft LF, Sampson MR, Thatcher N (2005) Randomized phase III trial of dose-dense chemotherapy supported by whole-blood hematopoietic progenitors in better-prognosis small-cell lung cancer. J Natl Cancer Inst 97: 666–674 15870437 10.1093/jnci/dji114

[bib27] Macleod PM, Tyrell CJ, Keeling DH (1988) The effect of cisplatin on renal function in patients with testicular tumours. Clin Radiol 39: 190–192 3128418 10.1016/s0009-9260(88)80022-9

[bib28] Motzer RJ, Nichols CJ, Margolin KA, Bacik J, Richardson PG, Vozelgang NJ, Bajorin DF, Lara PN, Einhorn L, Mazumdar M, Bosl GJ (2007) Phase III randomized trial of conventional-dose chemotherapy with or without high-dose chemotherapy and autologous hematopoietic stem-cell rescue as first-line treatment for patients with poor-prognosis metastatic germ cell tumors. J Clin Oncol 25: 247–256 17235042 10.1200/JCO.2005.05.4528

[bib29] Nichols CR, Catalano PJ, Crawford ED, Vogelzang NJ, Einhorn LH, Loehrer PJ (1998) Randomised comparison of cisplatin and etoposide and either bleomycin or ifosfamide in treatment of advanced disseminated germ cell tumours: an Eastern Cooperative Oncology Group, Southwest Oncology Group and Cancer and Leukaemia Group B Study. J Clin Oncol 16: 1287–1293 9552027 10.1200/JCO.1998.16.4.1287

[bib30] Nichols CR, Williams SD, Loehrer PJ, Greco FA, Crawford ED, Weetlaufer J, Miller ME, Bartolucci A, Schacter L, Einhorn LH (1991) Randomised study of Cisplatin dose intensity in poor-risk germ cell tumours: South Eastern Cancer Study Group and South West Oncology Group Protocol. J Clin Oncol 7: 1163–1172 10.1200/JCO.1991.9.7.1163 1710655

[bib31] O’Sullivan JM, Huddart RA, Norman AR, Nicholls J, Dearnaley DP, Horwich A (2003) Predicting the risk of bleomycin lung toxicity in patients with germ cell tumours. Ann Oncol 14: 91–96 12488299 10.1093/annonc/mdg020

[bib32] Osanto S, Bukman A, Van Hoek F, Sterk PJ, De Laat JA, Hermans J (1992) Long-term effects of chemotherapy in patients with testicular cancer. J Clin Onc 10(4): 574–579 10.1200/JCO.1992.10.4.574 1372350

[bib33] Pfreundschuh M, Trümper L, Kloess M, Schmits R, Feller AC, Rübe C, Rudolph C, Reiser M, Hossfeld DK, Eimermacher H, Hasenclever D, Schmitz N, Loeffler M, German High-Grade Non-Hodgkin's Lymphoma Study Group (2004) Two-weekly or 3-weekly CHOP chemotherapy with or without etoposide for the treatment of elderly patients with aggressive lymphomas: results of the NHL-B2 trial of the DSHNHL. Blood 104: 634–641 15016643 10.1182/blood-2003-06-2095

[bib34] Saxman SB, Nichols CR, Einhorn LH (1997) Pulmonary toxicity in patients with advanced-stage germ cell tumours receiving bleomycin with and without granulocyte colony stimulating factor (1997). Chest 111: 657–660 9118704 10.1378/chest.111.3.657

[bib35] Schmoll HJ, Kollmannsberger C, Metzner B, Hartmann JT, Schleucher N, Schöffski P, Schleicher J, Rick O, Beyer J, Hossfeld D, Kanz L, Berdel WE, Andreesen R, Bokemeyer C, German Testicular Cancer Study Group (2003) Long-term results of first-line sequential high-dose etoposide, ifosfamide, and cisplatin chemotherapy plus autologous stem cell support for patients with advanced metastatic germ cell cancer: an extended phase I/II study of the German Testicular Cancer Study Group. J Clin Oncol 21: 4083–4091 14568987 10.1200/JCO.2003.09.035

[bib36] Shamash J, Powles T, Ansell W, Berney D, Stebbing J, Mutsvangwa K, Wilson P, Asterling S, Liu S, Wyatt P, Joel SP, Oliver RT (2007b) GAMEC--a new intensive protocol for untreated poor prognosis and relapsed or refractory germ cell tumours. Br J Cancer 97: 308–314 17609665 10.1038/sj.bjc.6603865 PMC2360316

[bib37] Shamash J, Powles T, Mutsvangwa K, Wilson P, Ansell W, Walsh E, Berney D, Stebbing J, Oliver T (2007a) A phase II study using a topoisomerase I-based approach in patients with multiply relapsed germ-cell tumours. Ann Oncol 18: 925–930 17355956 10.1093/annonc/mdm002

[bib38] Thatcher N, Girling DJ, Hopwood P, Sambrook RJ, Qian W, Stephens RJ (2000) Improving survival without reducing quality of life in small – cell lung cancer patients by increasing the dose intensity of chemotherapy with granular site colony – stimulating factor support: results British Medical Research Council multicentre randomised trial. J Clin Oncol 18: 395–404 10637255 10.1200/JCO.2000.18.2.395

[bib39] Williams SD, Birch R, Einhorn LH, Irwin L, Greco FA, Loehrer PJ (1987) Treatment of disseminated germ-cell tumors with cisplatin, bleomycin, and either vinblastine or etoposide. New Eng J Med 316: 1435–1440 2437455 10.1056/NEJM198706043162302

